# Antiproliferative activity of methanolic extracts from two green algae, *Enteromorpha intestinalis* and *Rizoclonium riparium* on HeLa cells

**DOI:** 10.1186/2008-2231-21-72

**Published:** 2013-12-19

**Authors:** Subhabrata Paul, Rita Kundu

**Affiliations:** 1 Department of Botany, University of Calcutta, 35, Ballygunge Circular Road, Kolkata 700019, India

**Keywords:** *Enteromorpha intestinalis*, *Rhizoclonium riparium*, HeLa, MTT, Acridine orange, LC3B

## Abstract

**Background:**

Natural compounds can be alternative sources for finding new lead anti-cancer molecules. Marine algae have been a traditional source for bioactive compounds. *Enteromorpha intestinalis* and *Rhizoclonium riparium* are two well distributed saline/brackish water algae from Sundarbans. There’s no previous report of these two for their anti-proliferative activities.

**Methods:**

Cytotoxicity of the algal methanolic extracts (AMEs) on HeLa cells were assayed by 3-(4, 5-dimethylthiazol-2-yl)-2, 5- diphenyltetrazolium bromide (MTT) reduction assay. Morphological examinations were done by Haematoxylin, Hoechst 33258 and Acridine orange staining. DNA fragmentation was checked. Gene expressions of Cysteine aspartate protease (Caspase) 3, Tumor protein (TP) 53, Bcl-2 associated protein X (Bax) were studied by Reverse transcription- polymerase chain reaction (RT-PCR) keeping Glyceraldehyde 3-phosphate dehydrogenase (GAPDH) as internal control. Protein expressions were studied for Caspase 3, phospho-p53, Bax, Microtubule associated proteins-1/ light chain B (MAP1/LC3B) by western blot.

**Results:**

The AMEs were found to be cytotoxic with Inhibitory concentration 50 (IC50) values 309.048 ± 3.083 μg/ml and 506.081 ± 3.714 μg/ml for *E. intestinalis* and *R. riparium* extracts respectively. Treated cells became round with blebbings with condensed nuclei. Acidic lysosomal vacuoles formation occurred in treated cells. Expression of apoptotic genes in both mRNA and protein level was lowered. Expression of LC3B-II suggested occurrence of autophagy in treated cells.

**Conclusions:**

These two algae can be potent candidates for isolating new lead anticancer molecules. So they need further characterization at both molecular and structural levels.

## Background

Cancer is considered to be the second leading cause of death [1]. An estimated 12.7 million new cases are registered each year with 7.6 million deaths and 24.6 million persons living with cancer worldwide [2]. According to a recent report of World Health Organization (WHO), cervical cancer ranks as the most frequent cancer among women between 15–44 years age group in India and all over the world. Currently a population of more than 366 million women of India (age group of 15 or more), are at a risk of developing cervical cancer. Every year 529828 women are diagnosed with the disease and 275128 die from this cancer throughout the world. The scenario is not very different in India also. Here, each year, the estimated number of new cases is 134420 and number of mortality is 72825. Considering the growth rate of population, the projected new cases of cervical cancer by 2025 will be 720060 and projected number of death will be 395095 in the global context, whereas , in India, the projected new cases will be 115171 which is very alarming [3,4].

Chemotherapy is still the standard treatment method along with surgery and radiation therapy. Most of the available treatments cause severe side effects such as bleeding, cognitive impairment, sensory abnormalities, infertility, damage to hoemopoetic tissue, hair-loss due to their non-selective cytotoxicity. Emerging cancer drug resistance is another serious problem regarding chemotherapy [5]. Search for a new anticancer drug with lesser side effects and selective cytotoxicity has been one of the main thrust of cancer research worldwide. In this context, natural products, derived from plants, marine organisms and microorganisms, have drawn attentions of many scientists. According to WHO; 80% of world’s population, especially in developing countries rely on plant derived medicines [6]. In the few decades natural products from higher plants were explored in great extent for possible anticancer activities, while other groups (algae, fungi) were not given proper attention. But, there is a tremendous scope to obtain novel bioactive compounds from the algae. Algae are a promising group to furnish various bioactive compounds as they are a group of diverse (unicellular to multicellular, prokaryotic to eukaryotic) members with diverse (freshwater to brakish/marine) habitats. They have been traditionally used as food and medicine in India, China, Japan, Korea, Ireland and Wales. Brown seaweeds (macroalgae) like *Laminaria, Undaria* are utilized as sources of iodine [7]. Alginate produced by brown algae (seaweeds) is used in food and pharmaceutical industries due to its ability t*o* chelate metal ions. Carrageenans (water soluble sulfated galactans with an alternating backbone of α(1–4)-3,6 anhydro- D-galactose and ß(1–3) D galactose used as emulsifier/ stabilizers in milk based food products (ice-cream, pudding, desert gels, jams) are also obtained from macro algae (*Kappaphycus alvarezii)*[8]. Though, marine algae are rich source of pharmacologically active metabolites [9-13], having antimicrobial, antineoplastic, antiviral, anti-inflammatory and immunostimulant [14-16] activities, they were not exploited so far for their antiproliferating properties till today. Only few reports are available regarding the anticancer activities of algae. *Spirulina, Anabaena* and *Aphanizomenon*, members of Cyanophyceae (blue green algae) were reported to induce apoptosis in HL-60 and MCF7 cell lines [17-19]. But reports regarding the anticancer properties of Chlorophyta (green algae) are sparse. Extracts of *Udotea flabellum* was reported to have antiproliferative activity on HeLa, SiHa and KB cell lines [20].

In the present study, we have evaluated the cytotoxic potential of methanolic extracts of two green algae *Enteromorpha intestinalis* and *Rhizoclonium riparium,* using MTT assay on cervical cancer cell line (HeLa) followed by DNA fragmentation assay. Role of Caspase 3, p53 and Bax were studied in transcriptional and translational level to investigate their role in inducing cell death. Role of LC3B was studied at the translational level.

## Methods

### Chemicals and reagents

Methanol (Merck) was used to extract the algal materials. Dulbecco’s Modified Eagle Medium (DMEM), Fetal Bovine Serum (FBS) and antibiotic-antimycotic solution [Penicillin, Streptomycin & Amphotericin], Sodium bicarbonate (NaHCO_3_) (Himedia) were used in cell culture. 3-(4, 5-dimethylthiazol-2-yl)-2, 4-diphenyltetrazolium bromide (MTT) (Sigma) and Dimethyl sulfoxide (DMSO) (Merck) were used in cell viability assays. Haematoxylin (Gurr), Hoechst 33258 (Sigma), Paraformaldehyde (PFA)(HiMedia) were used in cellular and nuclear morphology study. Acridine orange (Sigma) was used in acidic vacuole staining. Non idet P-40 (NP40) (Sigma) was used in DNA isolation. Tris(hydroxymethyl)aminomethane (Tris), Ethylenediaminetetraacetic acid (EDTA), Acetic acid (Merck); Agarose, Ethidium bromide (EtBr) (Sigma) were used in Agarose gel electrophoresis. TRI reagent, Chloroform and Isopropanol (Sigma) were used for total RNA isolation. Sodium hypochlorite (NaClO) (Merck) used in RNA gel electrophoresis. First strand cDNA synthesis kit [MuLv RT kit] (Fermentas); Taq Polymerase, dNTPs (Chromus Biotech) were used in semi quantitative RT-PCR reactions. 100× protease inhibitor cocktail (G-Biosciences) was used at the time of protein isolation. Sodium hydroxide (NaOH), Sodium carbonate (Na_2_CO_3_), Sodium potassium tartrate (C_4_H_4_KNaO_6_.4H_2_O), Copper sulfate (CuSO_4_.5H_2_O) (Merck); Bovine serum albumin (BSA), Folin phenol reagent (Sigma) were used in protein quantification. Acrylamide, Bis-acrylamide, Ammonium persulfate (APS), Sodium dodecyl sulfate (SDS) (Merck) and Tetramethylethylenediamine (TEMED) (Sigma) were used in polyacrylamide gel electrophoresis (PAGE). Caspase3 (Imgenex); LC3B, ß-tubulin (Sigma); IgG-AP (Santa Cruz Biotechnology); Bax, phospho-p53 (Cell Signalling Technology); nitro-blue tetrazolium/5-bromo-4-chloro-3’-indolyphosphate (NBT/BCIP) (Sigma) and nitrocellulose paper (NC) (Schleicher & Schuell) were used in western blot analysis.

### Algal material

Two green algae, *Rhizoclonium riparium* (Roth) Harvey and *Enteromorpha intestinalis* (Linnaeus) [21] were collected during January 2012, from different blocks (Sandeshkhali and Jharkhali) of Sundarbans mangrove ecosystem (Indian part). Collected samples were properly identified and voucher specimens were deposited in the Calcutta University Herbarium (CUH). Accession numbers issued by CUH against *R. riparium* was CUH/AL/MW-48 and for *E. intestinalis* was CUH/AL/MW-50.

### Preparation of algal extracts

10 gm of algal samples were excised to small pieces and extracted with methanol (3 times) for 72 hours at room temperature. The extract was concentrated and dried with freeze dryer (Eyela). The dried mass was weighed and again dissolved in appropriate volume of methanol to make the concentration of the AMEs as 20 μg/μl. The extract was further filtered through a 0.22 μm sterile filter (Pall) and stored at −20°C for future use.

### Cell line

For the *in vitro* assay HeLa cell line was used. HeLa cells are Human Papilloma virus (HPV) 18 positive human cervical cancer cell line, expressing high risk E6 and E7 oncoproteins [22] and low level of p53. 293 T cells are normal human embryonic kidney (HEK) cell line expressing large T antigen of Simian virus 40 (SV-40), often used as transfection host.

### Cell culture

HeLa and 293 T cells were maintained in monolayer cultures in DMEM supplemented with 0.15% NaHCO_3_, 5% FBS, 2 mM L-glutamine, 0.45% L-glucose, 100units/ml penicillin, 100 μg/ml streptomycin and 250 ng/ml Amphotericin B at 37°C in a humidified incubator having 5% Carbon dioxide (CO_2_).

### MTT assay

Cytotoxicity of the AMEs was evaluated by standard MTT assay [23]. 2 × 10^4^ HeLa and 293 T cells /well were seeded in 96 well flat bottom culture plates along with negative control sets (without cells). After overnight incubation cells were treated with different concentrations (in a range of 0–500 μg/ml) of the AMEs in triplicate along with a vehicle control set (2.5% of the growth medium). After 24 hours, the medium with treatments were removed and replaced with fresh medium along with 100 μg of MTT in each well and incubated for another 4 hours. Then the medium was removed and the formazan crystals were dissolved in 100 μl DMSO and absorbance was taken at 490 nm [24] in an Elisa reader (BioRad). The extent of cytotoxicity was determined using the following formula:

$$\begin{array}{l} \mathrm{\%inhibition}=\left[1-\left(\mathrm{Absorbanc}e_{\mathrm{treated}}/\mathrm{Absorbanc}e_{\mathrm{non}-\mathrm{treated}}\right)\right]\\ \;\times 100 \end{array}$$

IC50 values were calculated subsequently. All the experiments were done with this IC50 doses.

### Morphological studies

AMEs treated HeLa cells were stained with Haematoxylin and Hoechst 33258 to study cellular and nuclear morphology. Treated and control cells were stained with supravital stain Acridine orange to study the formation of acidic vacuoles. 5x10^4^ HeLa cells/ coverslip were seeded and after overnight incubation, treated with IC50 values of the AMEs for 24 hours along with a non-treated set. Then fixed in 4% PFA and subsequently Haematoxylin and Hoechst staining were done.

For cellular morphology study, the fixed cells were stained with 1% acidic Mayer’s Haematoxylin for 5 minutes at room temperature. For nuclear morphology, 2 μg/ml of Hoechst 33258 was used to stain the nuclei for 15 minutes in dark at room temperature. For Acridine orange staining, after 24 hours of AME treatment, cells were additionally incubated in fresh media containing Acridine orange (1 μg/ml) for 15 minutes without fixation.

After the staining procedures were over, extra stain was drained out, washed briefly in phosphate buffered saline (PBS) and mounted in PBS containing 10% glycerol, 2% N-propyl gallate. Cells were visualized under both bright field and fluorescence {excitation 356 nm, emission 465 nm for Hoechst and excitation 488 nm, emission 530(green) 650(red) for Acridine orange} in an epi-fluorescence microscope (Olympus).

### DNA fragmentation

10^7^ HeLa cells were seeded in T25 flask. After overnight incubation, medium was replaced and fresh medium was added along with IC50 doses of AMEs. After 24 hours of treatment, cells were harvested and lysed with lysis buffer containing 20 mM EDTA, 50 mM Tris, 1%SDS and 1% NP-40 for 2 hours in ice. Then the total genomic DNA was purified and treated with RNase treatment (20 μg/ml) at 37°C for 1 hour. DNA was then precipitated, washed with 75% ethanol, briefly air-dried, dissolved in Tris-EDTA buffer (pH8) and electrophoresed in 1.5% agarose gel containing EtBr (0.5 μg/ml), Tris acetate EDTA (TAE) buffer at 4 V/cm, and subsequently visualized under ultra violet (UV) trans-illuminator and photographed in gel documentation system (UVP Multidoc-It).

### Gene and protein expression studies

10^7^ HeLa cells were seeded in T25 flask. After overnight incubation, medium was replaced and fresh medium was added along with IC50 doses of AMEs. After 24 hours of treatment, cells were harvested and RT-PCRs and Western blots were done.

### Semi quantitative RT-PCR

Total RNA was isolated from the cells using TRI reagent following manufacturer’s protocol. RNA integrity was checked in 1.5% agarose gel containing 0.3% NaClO [25], EtBr (0.5 μg/ml), TAE buffer at 4 V/cm with subsequent visualization under UV.

First strand cDNA synthesis was carried out taking 2 μg RNA using 1st strand cDNA synthesis kit [MuLv RT kit] following manufacturer’s protocol. Taking 2 μl of cDNA as template, PCRs were conducted for Caspase-3, Bax and TP53 gene while GAPDH serves as an internal control. The PCR products were subsequently subjected to agarose gel electrophoresis (AGE), visualized under ultra violet (UV) trans-illuminator and photographed in gel documentation system (UVP Multidoc-It). Densitometric analysis of the bands was done using UVP Doc-ItLS image analysis software.

The primers used were designed by Primer-3 software using FASTA sequences from NCBI-Nucleotide. The forward and reverse primer sequences along with their respective PCR profiles are listed in Table 1.

**Table 1:** Primer sequences and PCR profiles for the genes Caspase 3, Bax, TP53 and GAPDH

**A**	**Primer sequence (5’ → 3’)**						
Gene	Forward			Reverse			
Caspase 3	ATTGTGGAATTGATGCGTGA			GGCAGGCCTGAATAATGAAA			
TP53	ATGGCCATCTACAAGCAG			ACAGTCAAGAGCCAACCTCAG			
Bax	GTGGCAGCTGACATGTTTTC			GGAGGAAGTCCAATGTCCAG			
GAPDH	CAAGGTCATCCATGACAACTTTG			GTCCACCACCCTGTTGCTGTAG			
**B**	**PCR profile**						
PCR steps	Initial denaturation	Denaturation	Annealing	Extension	No. of cycles	Final extension	Product length (bp)
Gene							
Caspase 3	94°C – 4 m	94°C – 60s	52°C – 45 s	72°C – 60s	35	72°C – 7 m	205
TP53		94°C – 30s	58°C – 30s	72°C – 45 s			210
Bax		94°C – 60s	57°C – 30s	72°C – 45 s			151
GAPDH		94°C – 30s	58°C – 30s	72°C – 45 s			496

(A) Primer sequences, designed by Primer3 software tabulated.

(B) PCR profiles after optimization for single band for a target.

### Western blot

Total protein was isolated by lysing HeLa cells using 1xSDS gel loading buffer (50 mM Tris-Cl pH 6.8, 2% SDS, 10%2-Mercaptoethanol, 0.01% Bromophenol blue, 10% Glycerol) along with 1× protease inhibitor (with EDTA) followed by incubation in boiling water bath for 10 min. Then the lysates were centrifuged at 14000 g for 15 minutes at 4°C. Supernatants stored at −20°C for further uses.

Extracted protein samples were quantified following micro-Lowry method with slight modifications. To 2 μl of lysates 200 μl of 10% TCA was added and incubated 30 minutes at −20°C and the centrifuged at 15000 g for 15 minutes at 4°C. The precipitated protein pellets then re-dissolved in 100 μl of 1(N) NaOH and subsequently incubated in 1 ml Lowry reagent (2% Na_2_CO_3_:2% C_4_H_4_KNaO_6_.4H_2_O: 1% CuSO_4_.5H_2_O = 100:1:1) for 10 minutes at room temperature. Then 100 μl of Folin phenol reagent (diluted 1:1 with milli Q grade water) added to the samples and further incubated in dark at room temperature for 30 minutes. O.D of the samples was taken at 750 nm in a double beam spectrophotometer (Hitachi) and concentration of the samples was determined using a standard curve prepared with BSA.

100 μg of each protein samples were separated in 12% polyacrylamide gels. Semi dry transfer of the protein samples were done using 0.45 μm nitrocellulose paper, incubated in blocking buffer (5% BSA) for 2 hours at room temperature. Then the membrane was incubated at 4°C with appropriate dilutions of primary antibodies (1:2000 for anti ß-tubulin, 1:1000 for anti Bax, 1:500 for anti phospho-p53 and anti LC3B, 1:250 for anti Caspase 3) for overnight. After subsequent incubation of 2 hours with AP conjugated IgG (1:5000) at room temperature, NBT-BCIP solution was added and incubated until bands appeared. Pictures of the membranes were taken in gel documentation system (UVP Multidoc-It). Densitometry analysis of the bands was done using UVP Doc-ItLS image analysis software.

### Statistical analysis

All the experiments were carried out thrice in triplicates. All numerical data were expressed as mean of triplicates ± standard error (SE). Statistical analysis and generation of histograms were performed using Graphpad Prism 5.02 software. All datasets obtained from the experiments were subjected to grouped analysis by two-way analysis of variance (ANOVA) followed by Bonferroni post tests keeping p < 0.05 in all cases.

## Results

### MTT assay

**Figure 1:**
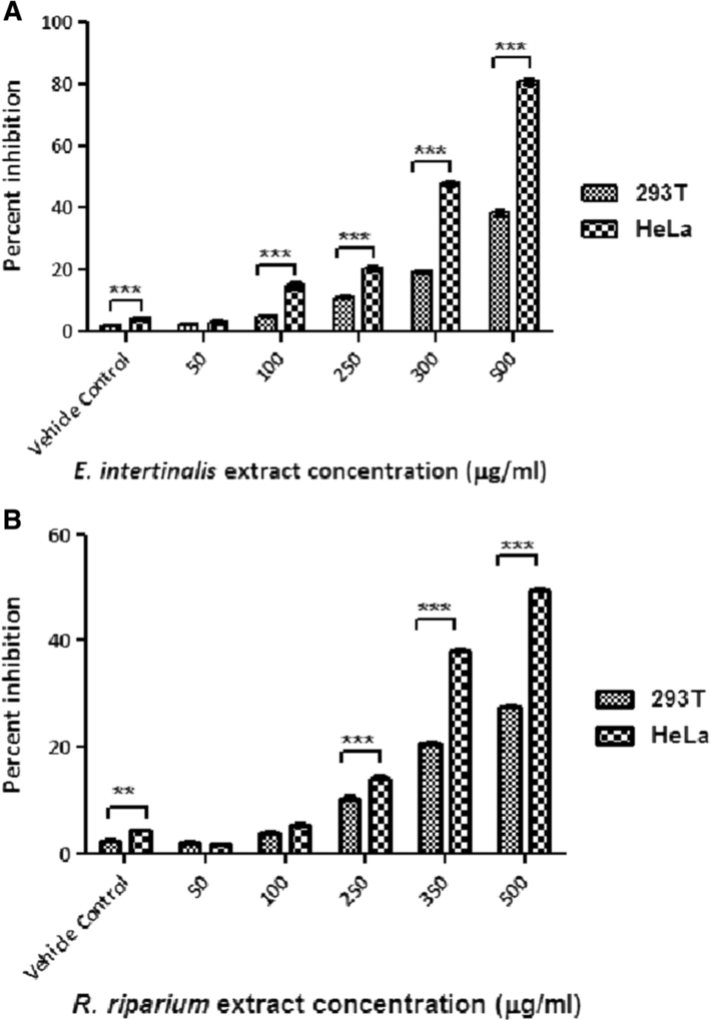
**Histograms showing the antiproliferative effects of the AMEs on HeLa and 293 T cell lines.** The cells were treated with increasing concentration of AMEs for 24 hours. Cytotoxicity of the AMEs was evaluated by MTT assay. Results showed percent inhibition of cell growth against increasing concentration of the extracts. **(A) ***E. intestinalis* extract treated, **(B) ***R. riparium* extract treated.Columns with bars represent mean ± SE of triplicates. Percent inhibitions of the AMES at each concentration point were compared between the two cells lines. According to the significance levels found, they were further categorized using various symbols as follows, **: p < 0.01; ***: p < 0.001.

To study the antiproliferative potential of the methanolic extract of these two algae on Hela cells and 293 T cells, MTT assays were done. Reduction of the substrate (MTT) by cellular dehydrogenase to form water insoluble formazan is the basis of the cytotoxicity test. Reduction in colour intensity in the treated sets than the non treated ones clearly indicated cytoxicity of the AME’s on HeLa cells. From the experiment it was observed that cell death was concentration dependent, number of nonviable cells increased with increased concentration of AMEs. We have observed that the IC50 doses of the AMEs in HeLa cells were 309.048 ± 3.083 μg/ml and 506.081 ± 3.714 μg/ml for *E. intestinalis* and *R. riparium* extracts respectively. For 293 T cells, the IC50 doses were 651.183 ± 1.198 μg/ml and 905.727 ± 4.034 μg/ml. The vehicle control set showed 4.345 ± 0.594 percent inhibition. From the study it was observed that methanolic extract of *E. intestinalis* was more cytotoxic than *R. riparium* (Figure 1a & b).

### Cell and nuclear morphology

**Figure 2:**
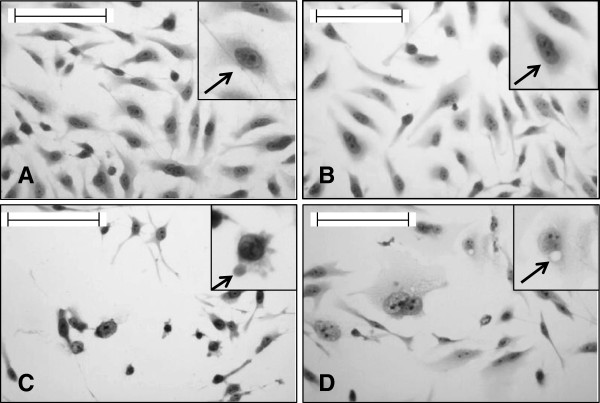
**HeLa cells grown on sterile cover slips were treated with IC50 doses of the AMEs for 24 hours.** Then they were stained with Haematoxylin to study the cellular morphology. Scale indicates 50 μm. Inset shows magnified view. **A)** Non-treated cells, the arrow shows normal cellular morphology **B)** Vehicle control cells, the arrow shows normal cellular morphology **C)** Cells treated with *E. intestinalis* extract, the arrow shows cytoplasmic blebbings **(D)** Cells treated with *R. riparium* extract, the arrow shows cytoplasmic vacuolation.

**Figure 3:**
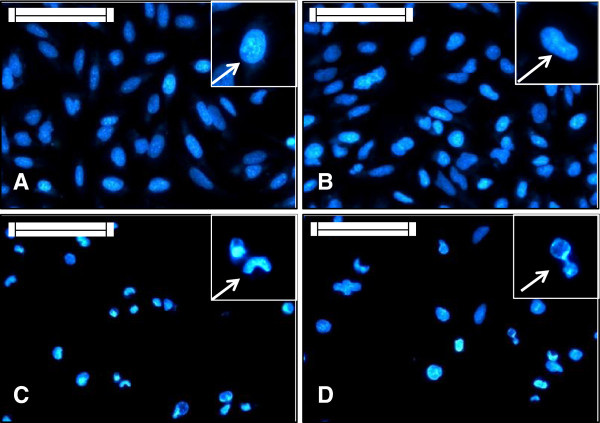
**HeLa cells grown on sterile cover slips were treated with IC50 doses of the AMEs for 24 hours.** Then they were stained with Hoechst 33258 to study the nuclear morphology. Scale indicates 50 μm. Inset shows magnified view. **(A)** Non-treated cells, the arrow shows normal looking nucleus; **(B)** Vehicle control cells, the arrow shows normal elliptical nucleus; **(C) ***E. intestinalis* extract treated sets, the arrow shows nuclei with deformity; **(D) ***R. riparium* treated cells, the arrow shows highly condensed nuclei.

Both the AMEs treated HeLa cells showed distinct differences in their cellular and nuclear morphology in comparison to the untreated cells. In the treated sets, cells became round, lost their characteristic stretched appearance, showed clear cytoplasmic blebbings and vacuolation. Cells stained with Hoechst, observed under fluorescence microscope showed nuclear morphology. Nuclei of the control sets were elliptical in shape, but the treated nuclei were somewhat different. In the treated cells nuclear condensation was pronounced with deformed appearances. Vehicle sets in both cases showed characteristic morphological features as the non-treated ones (Figures 2 and 3).

### Acidic vacuole localization

**Figure 4:**
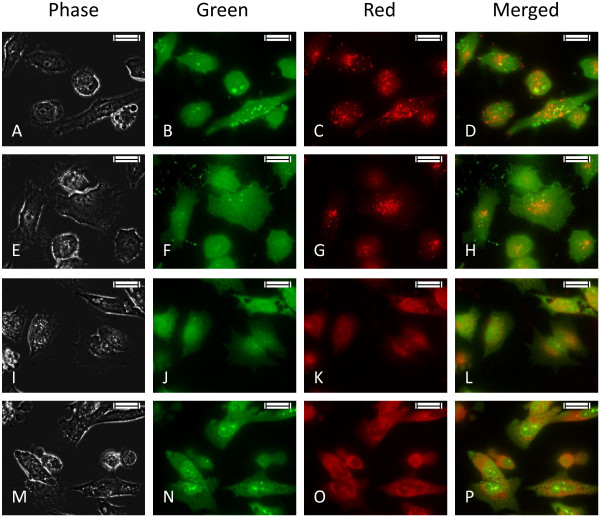
**HeLa cells grown on sterile cover slips were treated with IC50 doses of AMEs for 24 hours.** Then they were stained with Acridine orange (1 μg/ml) to study the formation of acidic vacuoles. Scale indicates 50 μm. **A-D)** Non-treated, **E-H)** Vehicle control, **I-L) ***E. intestinalis* extract treated, **M-P) ***R. riparium* treated HeLa cells.

Acridine orange in uncharged form, stain both cytoplasm and nucleic acids which fluoresce bright green. Whereas in protonated form it accumulates in the lysosomal acidic vacuoles, form aggregates and fluoresce bright red [26].When studied under a fluorescence microscope, it was observed that in the non-treated and vehicle control sets minimal red fluorescence was found, whereas, increased red fluorescence were observed in the treated cells. In *R. riparium* extract treated cells almost the whole cytoplasm became red (Figure 4) indicating the merging of all the acidic vacuoles.

### DNA fragmentation assay

**Figure 5:**
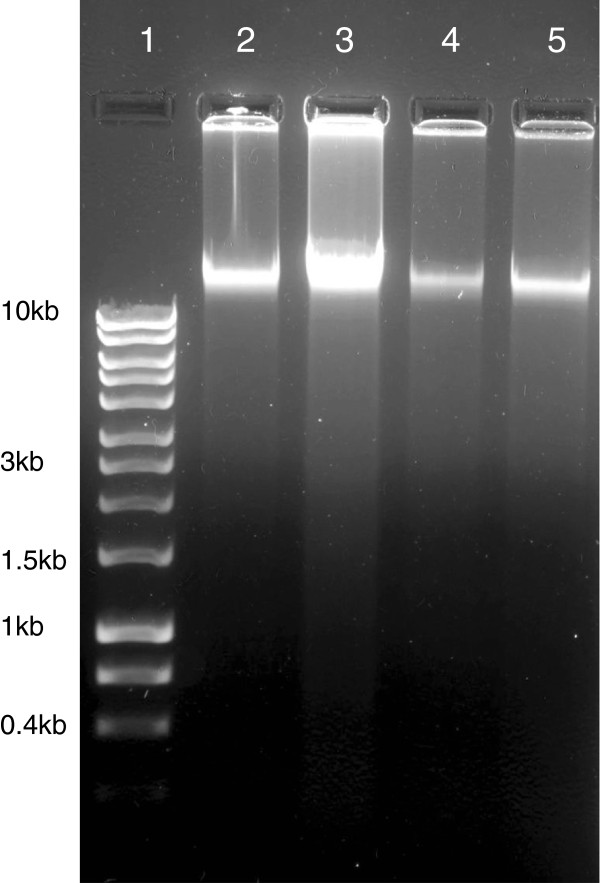
**HeLa cells grown in T25 flask were treated with IC50 doses of AMEs for 24 hours along with a control set.** Genomic DNA was isolated from all the sets and electrophorased in 1.5% agarose gel. Lane **1)** Molecular marker (0.4-10 kb, Lane **2)** Non-treated, Lane **3)** Vehicle control, Lane **4) ***E. intestinalis* extract treated, Lane **5) ***R. riparium* extract treated.

DNA fragmentation is a classical hallmark of apoptosis. But in the treated cells no sign of DNA laddering was observed (Figure 5). Intact genomic DNA was found in all the cells which implied that apoptosis might not be the mechanism for inducing cell death in the treated ones.

### Semi-Q RT-PCR

**Figure 6:**
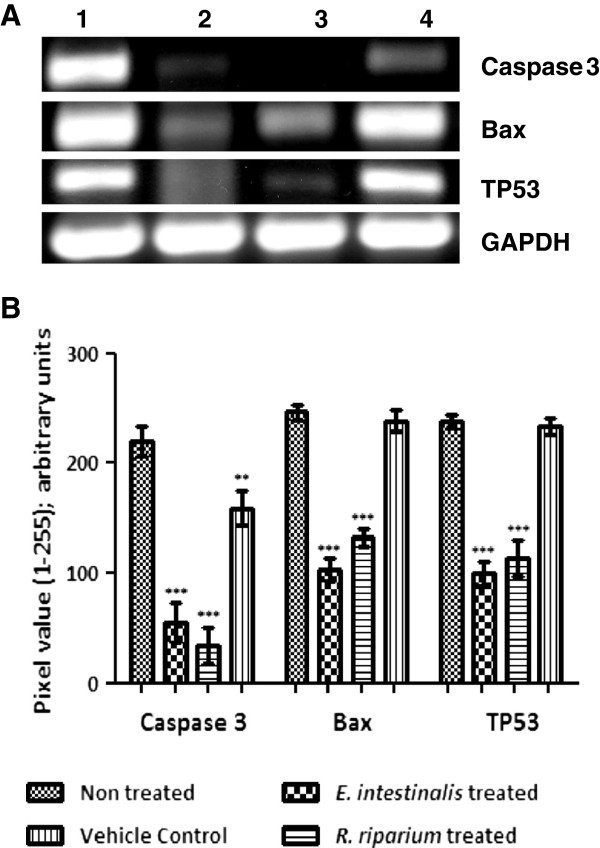
**Gene expression profiles of Caspase 3, Bax, TP53, GAPDH in HeLa cells treated with AMEs (IC50 doses) by semi quantitative RT-PCR. (A)** Representative pictures of three independent experiments. Lane 1) Non-treated, Lane 2) *E. intestinalis* extract treated, Lane 3) *R. riparium* treated, 4) Vehicle control. **(B)** Relative levels of gene expressions after normalization for GAPDH were determined by densitometry analysis of the bands, represented by histogram. Columns with bars represent mean ± SE of triplicate pixel values for the bands. Intensity for each band in the treated sets was compared with that of in non treated sets. According to the significance levels found, they were further categorized using various symbols as follows, **: p < 0.01; ***: p < 0.001.

To investigate the role of Caspase 3, Bax and p53 in AMEs induced cell death in HeLa cells, expression profile of these genes were studied by semi q RT-PCR. For the gene expression studies, RT-PCR gave us a qualitative estimation of up regulation/down regulation of some apoptotic genes (Figure 6). Caspase 3 expression was found to be downregulated in both *E. intestinalis* treated (75.45%) and *R. riparium* treated (85.54%) cells. Bax expression was down regulated in both the treated samples, which was more pronounced in *E. intestinalis* (58.13%) extract treated cells than *R. riparium* (46.34%) treated cells. TP53 expression also decreased in both the samples, slightly higher in *E. intestinalis* (58.22%) extract treated cells than *R. riparium* (53.22%) treated cells. The down regulated expression profiles of caspase 3 and Bax in the treated cells indicate that cell death was not mediated by Bax and caspase3.

### Immunoblotting

**Figure 7:**
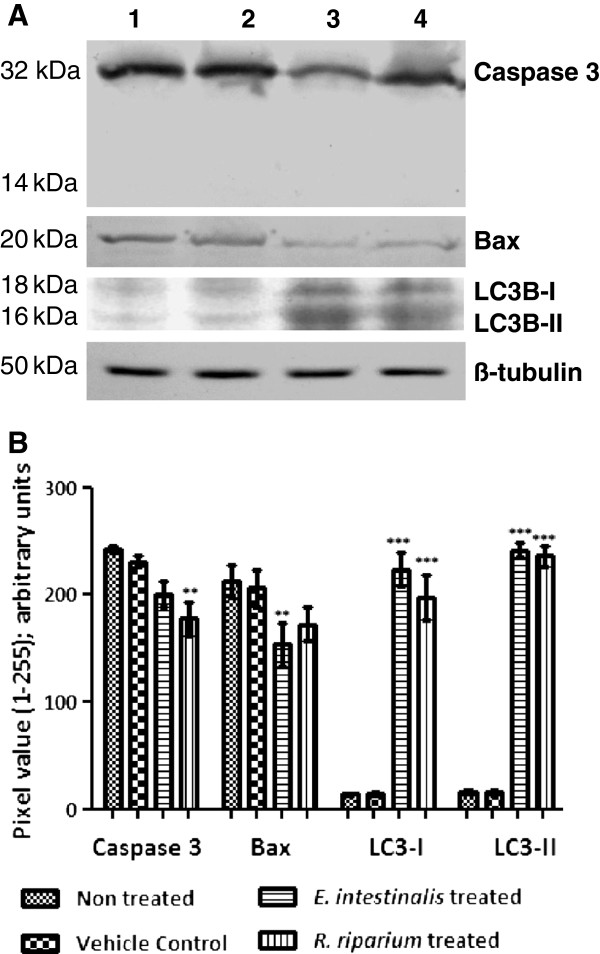
**Protein expression profiles of Caspase 3, Bax, LC3B-I & II and ß-tubulin in HeLa cells treated with AMEs (IC50 doses) by Western blot. (A)** Representative pictures of three independent experiments. Lane 1) Non-treated, Lane 2) Vehicle control 3) *E. intestinalis* extract treated, Lane 4) *R. riparium* treated. **(B)** Relative levels of gene expressions after normalization for ß-tubulin were determined by densitometry analysis of the bands, represented by histogram. Columns with bars represent mean ± SE of triplicate pixel values for the bands. Intensity for each band in the treated sets was compared with that of in non treated sets. According to the significance levels found, they were further categorized using various symbols as follows, **: p < 0.01; ***: p < 0.001.

Expression level of some apoptotic and autophagic proteins were monitored at the translational level (Figure 7). In all the samples cleaved caspase3 expression was absent while Pro-Caspase 3 expression was less down regulated in *E. intestinalis* (17.35%) than *R. riparium* (26.85%) treated cells. Bax expression on the other hand was found to be more down regulated in *E. intestinalis* (27.83%) than *R. riparium* (18.86%) treated cells. Expression of phospho-p53 was absent in all the samples. Expression of LC3B-I and LC3B-II was found in both the treated samples while absent in non-treated and vehicle control sets. LC3B-I and LC3B-II expressions were higher (8.07% and 19.79% respectively) in *R. riparium* extract treated cells than the *E. intestinalis* treated cells.

## Discussion

In this study, we have evaluated the methanolic extracts of two green algae, *E. intestinalis* and *R. riparium* for their antiproliferative activity in human cervical cancer cell line HeLa. These two green algae are found widely in the water bodies of Sundarbans mangrove ecosystem and commonly used as fish feed. There were no previous reports about their antiproliferative property on cervical cancer cell lines. An alkali extracted polysachharide (DAEB) from *E. intestinalis*is was found to be capable of preventing formation of Sarcoma 180 tumor and had some antitumor activity which was mediated by immunoenhancement of immune system instead of direct cytotoxicity [27].

To determine the IC50 doses of these two AMEs, MTT assays were done. Along with HeLa cells, a non-cancerous cell line, 293 T (human embryonic kidney cell line) were treated with the AMEs. From the results, it was observed that IC50 doses of the AMEs were significantly much higher for the 293 T cell line that the HeLa cell line. Which indicates that the AMEs were less cytotoxic to the non-cancerous cell line. Grouped analysis using Two-way ANOVA followed by Bonferroni posttests revealed that with respect to the control 293 T cells cytotoxicity of the AMEs in HeLa cells were significant above 50 μg/ml for *E. intestinalis* extract and above 100 μg/ml for *R.riparium* extract. This implies that *E.intestinalis* extract is more potent than *R.riparium* extract.

Cellular and nuclear morphology observed in the treated cells showed clear indication of cytotoxicity. But the responses were different for the two extracts. In *E. intestinalis* extract treated cells, cytoplasmic blebbings were observed along with deformed nuclei, while cytoplasmic vacuolation with condensed nuclei was observed in *R. riparium* treated cells. Chromatin condensations into compact figures, which are often globular or crescent shaped, are defined as stage II chromatin condensation, and occurs in apoptosis. Cytoplasmic blebbings are generally associated with apoptosis where cells undergoing apoptosis breaks down into apotosomes/apoptotic bodies, which were further absorbed by macrophages (*in vivo*), whereas cytoplasmic vacuolation is associated with autophagic cell death, a type II programmed cell death. During autophagy, portions of the cytoplasm and subcellular organelles are sequestered by endoplasmic reticulum, resulting in vesicular bodies which acted as autophagosomes. They are further fused with the lysosomes to form autophagosomal vesicles where the contents are enzymatically degraded [28]. For detecting the acidic compartments, lysosomotropic agent acridine orange was used for staining. Cells treated with *R. riparium* extract showed higher amount of acidic vacuole formation. On the other hand cells treated with *E. intestinalis* extract showed lesser number of acidic vacuole formation. In *R. riparium* treated cells, all most the whole cell became red, due to merging of all the acidic vacuoles. The observation was further validated by immunoblot results.

DNA fragmentation is a classical hallmark of apoptosis. In response to apoptotic signals (DNA damage/ stress) proapoptotic Bcl2 family protein Bax becomes activated resulting in mitochondrial membrane permeabilization. As a result cytochrome C and APAF-1 (apoptotic protease activating factor 1) are released from the inter-membrane space and activate caspase 9 through cleavage. Caspase 9 generates a signaling cascade of caspase cleavage that results in DNA fragmentation into 180 basepairs and multiples of it. The effecter caspase is caspase 3. p53 has a major role in cell survival . In healthy cells the nuclear amount of p53 is very low. Due to binding of adapter protein MDM2, p53 is subsequently exported to cytosol and degraded. Whereas in the cells with damaged DNA, p53 becomes phosphorylated. As MDM2 cannot recognize phosphorylated p53, the nuclear p53 is stabilized and induce pro-apoptotic proteins (Bax, Puma and Noxa etc.). We have found some signs of apoptotic features (nuclear and cellular) in the AMEs treated cells, so we have undertaken the DNA fragmentation assay and the same time assessed the expressions of caspase 3, Bax and p53 at the transcriptional level. From our observation we found that DNA fragmentation was absent indicating a cell death process not mediated by caspase 3 or Bax.

RT-PCR observation also validates the findings. Here Bax and caspase 3 expressions were found to be less in comparison to the controlled cells and expression of p53 was down regulated in all the samples. In translational levels, the expression of cleaved pro-caspase 3 and Bax were found to be down regulated in both the samples whereas no expressions of cleaved caspase 3 and phospho-p53 (data not shown) were observed. Absence of DNA fragmentation, down regulation of Bax, p53 and absence of cleaved caspase 3 strongly indicated a cell death pathway other than apoptosis or type I cell death.

As the AMEs treated samples showed some signs positive (vacuolation in cytosol) for autophagy or type-II cell death, role of LC3 was studied by western blot. LC3 (MAP1) is a mammalian homolog of the yeast ATG8 protein, a ubiquitin like protein that becomes lipidated and tightly associated with autophagosomal membranes. LC3 proteins are specifically cleaved at their carboxy terminal to form LC3-I, which has an exposed carboxy terminal glycine that is conjugated to phosphatidylethanolamine to form LC3-II. This LC3-II protein bounds tightly with the autophagosomal membranes and serves as an autophagic marker protein [29]. Our results clearly showed the presence of LC3-I in both the treated samples, while the presence of LC3-II was distinct in *R. riparium* treated samples. All these observations strongly suggest that these two algal extracts are capable of inducing cell death by autophagy. Previously autophagy was considered as a pro-survival mechanism of the cell. But recent studies suggest that autophagy result in cell deaths and sometimes activates apoptosis.

## Conclusions

From the results it was quite evident that, both the AMEs had potent cytotoxicity on HeLa cells. With the lesser IC50 value, *E. intestinalis* extract was found to be more antiproliferative (usage of Methanol as vehicle exert negligible amount of cytotoxicity to the cells). The cellular morphological examinations revealed presence of vacuoles which may be due to formation of auto lysosomal vacuoles. In case of nuclear morphology study, though nuclear condensation was observed, absence of fragmented nuclei along with absence of DNA laddering indicated that any other mechanism than apoptosis is responsible for the cell death. Whereas increase in acidic vacuoles and expression of LC3B-II had suggested autophagic cell death in the treated samples.

Both the *E. intestinalis* and *R. riparium* methanolic extracts were found to be cytotoxic to HeLa cells, most probably by the involvement of autophagy. So they were potent candidates for further characterization of their chemical constituents and the molecular pathway by which they worked.

## Abbreviations

AGE: Agarose gel electrophoresis; AME: Algal methanolic extract; APS: Ammonium persulfate; Bax: Bcl-2 associated protein X; BSA: Bovine serum albumin; C4H4KNaO6.4H2O: Sodium potassium tartrate; Caspase 3: Cysteine aspartate protease 3; CUH: Calcutta university herbarium; CuSO4.5H2O: Copper sulfate; DMEM: Dulbecco’s modified eagle medium; DMSO: Dimethyl sulfoxide; EDTA: Ethylenediaminetetraacetic acid; EtBr: Ethidium bromide; FBS: Fetal bovine serum; GAPDH: Glyceraldehyde 3-phosphate dehydrogenase; HPV: Human papilloma virus; IC50: Inhibitory concentration 50; MAP1/LC3B: Microtubule associated proteins-1/ light chain B; MTT: 3-(4, 5-dimethylthiazol-2-yl)-2, 5- diphenyltetrazolium bromide; Na2CO3: Sodium bicarbonate; NaClO: Sodium hypochlorite; NaHCO3: Sodium bicarbonate; NaOH: Sodium hydroxide; NBT/BCIP: Nitro-blue tetrazolium/5-bromo-4-chloro-3’-indolyphosphate; NC: Nitrocellulose; NP-40: Non idet P40; PAGE: Polyacrylamide gel electrophoresis; PBS: Phosphate buffered saline; PFA: Paraformaldehyde; RT-PCR: Reverse transcription- polymerase chain reaction; SE: Standard error; SDS: Sodium dodecyl sulfate; TAE: Tris acetate EDTA buffer; TCA: Trichloro acetic acid; TEMED: Tetramethylethylenediamine; TP53: Tumor protein 53; Tris: Tris(hydroxymethyl)aminomethane; WHO: World health organization.

## Competing interest

The authors declare that they have no competing interest.

## Author’s contributions

SP: Collected algae, prepared extracts and performed all the experiments; RK: conceived the experiment, and participated in its design and coordination and helped to draft the manuscript. All authors read and approved the final manuscript.

## References

[B1] Xu H Yao L Sung H Wu L Chemical composition and antitumor activity of different polysaccharides from the roots Actinidia eriantha Carbohydr Pol 2009 21 316 322 10.1016/j.carbpol.2009.04.007

[B2] Jemal A Bray F Center MM Ferlay J Ward E Forman D Global cancer statistics Ca Cancer J Clin 2011 21 69 90 21296855 10.3322/caac.20107

[B3] WHO/ICO Information Centre on HPV and Cervical Cancer (HPV Information Centre). Human Papillomavirus and Related Cancers in India. Summary Report 2010 http://www.hpvcentre.net/statistics/reports/IND.pdf

[B4] Globocan 2008 http://globocan.iarc.fr/pages/fact_sheets_cancer.aspx

[B5] Gottesman MM Mechanisms of cancer drug resistance Annu Rev Med 2002 21 615 627 10.1146/annurev.med.53.082901.103929 11818492

[B6] Gurib-Fakim A Medicinal plants: traditions of yesterday and drugs of tomorrow Mol Aspects Med 2006 21 1 1 93 10.1016/j.mam.2005.07.008 16105678

[B7] Boopathy NS Kathiresan K Anticancer drugs from marine flora: an overview J Oncol 2010 21 1 18 10.1155/2010/214186 PMC3065217 21461373

[B8] Cardazo KHM Guaratini T Barros MP Falcao VR Tonon AP Lopes NP Campos S Torres MA Souza AO Colepicolo P Pinto E Metabolites from algae with economic impact Comparative Biochemistry and Physiology Part C 2007 21 60 78 10.1016/j.cbpb.2006.09.003 16901759

[B9] Cannell JPR Algae as a source of biologically active products Pestic Sci 1993 21 147 153 10.1002/ps.2780390208

[B10] Nekhoroshev MV The black sea algae are potential source of antitumor drugs Al’-gologiya 1996 21 1 86 90

[B11] Mayer A Lehmann V Marine pharmacology in 1999: antitumor and cytotoxic compounds Anticancer Res 2001 21 2489 2500 11724312

[B12] Mayer AMS Gustafson KR Marine pharmacology in: antitumor and cytotoxic compounds Int J Cancer 2000 21 105 291 299 10.1002/ijc.11080 12704660

[B13] Faulkner DJ Marine natural products Nat Prod Rep 2000 21 7 55 10.1039/a809395d 10714898

[B14] Rinehart KL Jr Shaw PD Shield LS Gloer JB Harbour GC Koker MES Samain D Schwartz RE Tymiak AA Weller DL Carter GT Munro MHG Marine natural products as sources of antiviral, antimicrobial, and antineoplastic agents Pure and Appl Chem 1981 21 795 871 10.1351/pac198153040795

[B15] Tziveleka LA Vagias C Roussis V Natural products with anti-HIV activity from marine organisms Curr Top Med Chem 2003 21 13 1512 1535 10.2174/1568026033451790 14529524

[B16] Konig GM Wright AD Marine natural products research. Current directions and future potential Planta Med 1995 21 193 211 10.1055/s-2006-957861 8693030

[B17] Bechelli J Coppage M Rosell K Liesveld J Cytotoxicity of algae extracts on normal and malignant cells Leuk Res Treatment 2011 21 Article ID 373519, 7 pages 10.4061/2011/373519 PMC3505942 23213541

[B18] Li B Chu X Gao M Li W Apoptotic mechanism of MCF-7 breast cells in vivo and in vitro induced by photodynamic therapy with C-phycocyanin Acta Biochim Biophys Sin 2010 21 80 89 10.1093/abbs/gmp104 20043050

[B19] Oftedal L Selheim F Wahlsten M Sivonen K Døskeland SO Herfindal L Marine benthic cyanobacteria contain apoptosis-inducing activity synergizing with daunorubicin to kill leukemia cells, but not cardiomyocytes Mar Drugs 2010 21 2659 2672 10.3390/md8102659 21116413 PMC2992999

[B20] Moo-puck R Robledo D Fredle-Pelegrin Y In vitro cytotoxic and antiproliferative activities of marine macroalgae from Yucatan, Maxico Ciencias Marinas 2009 21 4 345 358

[B21] Algaebasehttp://www.algaebase.org

[B22] DeFilippis RA Goodwin EC Wu L DiMaio D Endogenous human papillomavirus E6 and E7 proteins differentially regulate proliferation, senescence, and apoptosis in HeLa cervical carcinoma cells J Virol 2003 21 1551 1563 10.1128/JVI.77.2.1551-1563.2003 12502868 PMC140828

[B23] Carmicheal J DeGraff WG Gazder AF Evaluation of a tetrazolium-based semiautomated colorimetric assay: assessment of chemosensitivity testing Cancer Res 1987 21 936 942 3802100

[B24] Pang M Gao Wu Z Lv N Wang Z Tang X Qu P Apoptosis induced by yessotoxins in Hela human cervical cancer cells in vitro Molecular Medicine Reports 2010 21 629 634 21472289 10.3892/mmr_00000307

[B25] Aranda PS LaJoie DM Jorcyk CL Bleach gel: a simple agarose gel for analyzing RNA quality Electrophoresis 2002 21 366 369 10.1002/elps.201100335 PMC3699176 22222980

[B26] Paglin S Hollister T Delohery T Hackett N McMahill M Sphicas E Domingo D Yahalom J A novel response of cancer cells to radiation involves autophagy and formation of acidic vacicles Cancer Res 2001 21 439 444 11212227

[B27] Jiao L Li X Li T Jiang P Wu M Zhang L Characterization and anti-tumor activity of alkali-extracted polysaccharide from *Enteromorpha intestinalis* Int Immunopharmacol 2009 21 324 329 10.1016/j.intimp.2008.12.010 19159698

[B28] Yang Y Liang Z Gu Z Qin Z Molecular mechanism and regulation of autophagy Acta Pharmacol Sin 2005 21 12 1421 1434 10.1111/j.1745-7254.2005.00235.x 16297339

[B29] Hansen TE Johansen T Following autophagy step by step BMC Biol 2011 21 39 10.1186/1741-7007-9-39 21635796 PMC3107173

